# Assessment of Stress Distribution and Displacement in the Craniofacial Complex between Facemask Therapy and Bone Anchored Maxillary Protraction (BAMP): A 3D Finite Element Study

**DOI:** 10.12688/f1000research.173765.1

**Published:** 2025-12-22

**Authors:** Sandra Susan Saji, Dilip Kumar N, Prakash A.T, Shruthi M S, Usha G V, Bhuvaneshwari Nadar, Sultan Almalki, Khalid Gufran, Inderjit M. Gowdar

**Affiliations:** 1 Orthodontics and Dentofacial Orthopedics, Bapuji Educational Association College of Dental Sciences, Davangere, Karnataka, India; 2 Public Health Dentistry, Bapuji Dental College and Hospital, Davangere, Karnataka, India; 3 Public Health Dentistry, Terna Dental College and Hospital, Navi Mumbai, Maharashtra, India; 4 Preventive Dental Sciences, Prince Sattam bin Abdulaziz University College of Dentistry, Al Kharj, Riyadh Province, Saudi Arabia; 5 Prothodontics, Prince Sattam bin Abdulaziz University College of Dentistry, Al Kharj, Riyadh Province, Saudi Arabia

**Keywords:** Facemask, Bone Anchored Maxillary Protraction, Finite Element Analysis, Class III malocclusion

## Abstract

**Background:**

Class III malocclusion presents unique challenges in both diagnosis and treatment. Bone-anchored maxillary protraction (BAMP) and facemask (FM) therapy are common approaches. Finite element analysis (FEA) helps evaluate their biomechanical effects. The study aims to evaluate and compare the stress distribution and displacement during maxillary protraction on the craniofacial complex using bone-anchored maxillary protraction and a facemask using finite element analysis.

**Methods:**

A finite element model of the maxillofacial complex was developed using CT scans of a young human skull and analyzed using ANSYS (v18.1). The material properties for facemask therapy and miniplates used in bone-anchored maxillary protraction (BAMP), were gathered to create a 3D finite element analysis (FEA) model. Displacement and stress patterns were assessed in the maxilla, maxillary teeth, mandible, and surrounding structures for both BAMP and facemask therapy, applying a bilateral force of 300 grams.

**Results:**

Displacement was mainly observed in the maxillary complex for both protocols, with BAMP showing more anterior displacement than the facemask. In contrast, the mandible experienced greater downward and backward displacement with the facemask protocol. Both methods revealed distinct displacement patterns in the dentoalveolar area. The facemask treatment resulted in more forward displacement of the maxillary anterior teeth, while BAMP primarily caused forward displacement in the first and second molar regions. Additionally, BAMP showed higher stress levels in the craniofacial area, particularly at the miniplate attachment site, whereas the Facemask approach had a more even distribution of stress across the craniofacial region.

**Conclusion:**

This finite element analysis indicates that maxillary protraction can be achieved through both FM and BAMP methods. However, BAMP method showed greater skeletal displacement with fewer dental effects. This implies that skeletal anchorage might provide a better result for treating class III malocclusion.

## Introduction

A Class III malocclusion is generally straightforward to diagnose but presents a significant challenge in terms of treatment. Early intervention is crucial, as it can help guide skeletal growth and potentially avoid surgical options later on. Orthopaedic appliances play a key role in managing this condition, especially in growing patients. The protraction facemask is particularly effective for encouraging forward growth of the maxilla. It works by applying anterior and downward forces, which can help improve the relationship between the maxilla and mandible. Other appliances, like the chin cup and reverse twin block, are designed to alter mandibular positioning or encourage maxillary advancement. The Frankel Regulator-3 can also be beneficial by improving the arch form and allowing for more harmonious dental relationships. Each appliance has its indications and can be chosen based on the individual’s specific skeletal and dental needs. Early diagnosis and appropriate use of these appliances can lead to more favorable outcomes, potentially reducing the need for later surgical intervention. Regular follow-up and adjustments are essential to monitor progress and make necessary modifications throughout treatment. ^1^

The protraction facemask is effective in facilitating anterior movement of the maxilla, leading to improvements in the dental and skeletal relationships in Class III malocclusion. By applying anterior forces, the facemask encourages forward growth of the maxilla, helping to improve skeletal relationships. This can enhance the alignment and positioning of the upper teeth, contributing to better occlusion. This can aid in achieving a more favorable incisal relationship. The appliance can help in positioning the mandible more favorably in relation to the maxilla. However, reliance on the dentition for force transmission can lead to several unintended consequences, such as retroclination of lower incisors, proclination of upper incisors, mesial movement with extrusion of upper molars, clockwise mandibular rotation, and increased lower facial third dimension. ^2^ Despite these challenges, careful management and monitoring during treatment can help mitigate some of these unwanted effects, ensuring a more balanced outcome for the patient. ^3, 4^ Adjustments to the treatment plan may be necessary based on individual responses to the appliance.

To address the limitations associated with traditional facemask therapy, skeletal anchorage-based approaches like bone-anchored maxillary protraction (BAMP) have emerged. Introduced by De Clerck et al. in 2009, BAMP utilizes mini plates anchored to the bone, allowing for more effective maxillary advancement while minimizing dentoalveolar side effects. ^5^ BAMP has certain key features, such as intraoral elastics, which are attached to the mini plates, providing a direct means of applying force to the maxilla without relying on the dentition for anchorage. Reduced dentoalveolar effects; the skeletal anchorage allows for more precise control over maxillary positioning, facilitating effective protraction, and improved aesthetic outcomes. BAMP represents a significant advancement in the treatment of Class III malocclusion, providing orthodontists with a powerful tool to achieve desired skeletal changes while preserving dental relationships. ^6^ As with any treatment, careful planning and monitoring are essential to ensure optimal results for each individual patient.

While bone-anchored maxillary protraction (BAMP) offers significant advantages in terms of minimizing dentoalveolar side effects and improving skeletal outcomes, it does come with notable disadvantages. The need for surgical placement and subsequent removal of the mini plates introduces inherent risks associated with any surgical procedure, such as infection, bleeding, and anesthesia complications. Additional drawbacks include postoperative inflammation, potential irritation of adjacent tissues by the mini plates or elastics, and the risk of miniplate loosening due to inadequate bone quality at a young age. ^7^ Analyzing displacement and stress distribution in the craniofacial complex using 3D finite element analysis (FEA) can provide crucial insights into the effectiveness and biomechanics of different treatment modalities, such as bone-anchored maxillary protraction (BAMP) and facemask therapy. The study aims to evaluate and compare the stress distribution and displacement on various craniofacial structures caused during bone-anchored maxillary protraction (BAMP) and facemask therapy using the finite element method (FEM).

## Materials and methods

**Figure 1.**
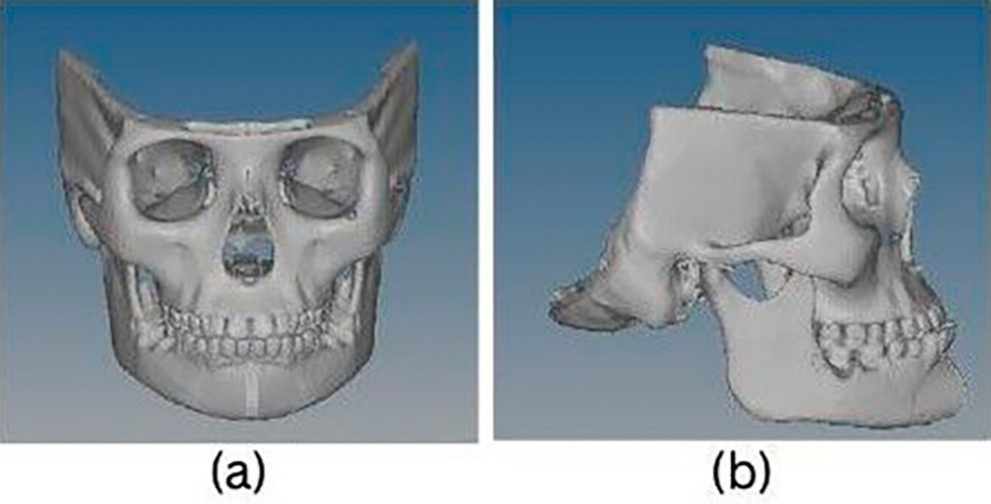
(a and b): CT scan of a skull with Class III malocclusion seen in (a) frontal view and (b) lateral view.

Pre-existing CT image ( Figure 1a and b) of a dry skull with prognathic mandible and retrognathic maxilla causing Class III malocclusion at an age range of 11 to 12 years was acquired. In Materialise’s Interactive Medical Image Control System (MIMIC Version 20.0), the DICOM raw data from the CT scan was segmented to extract the skeletal features. After segmenting the skull, surface mesh was created. The FEM analysis was performed using the exported meshed models into the ANSYS (version 18.1) software.

Finite element analysis is carried out in three different stages:

1. Pre-processing, 2. Solution, 3. Post-processing

**Pre-processing**

The 3D skull is transformed into a finite element model in this initial step of the process, and material properties are assigned. The surface mesh from MIMIC was imported during pre-processing, and it was transformed into a 3D finite element mesh with linear tetrahedron elements and given material attributes. The cortical bone, cancellous bone, titanium, and stainless-steel material qualities were used in the manner specified in Table 1.

**Table 1.** Elastic modulus and Poisson’s ratio of materials of the models.

Material components	Elastic modulus (GPa)	Poisson’s ratio
Cortical bone	13.7	0.30
Cancellous bone	1.37	0.30
Stainless Steel	200	0.30
ABS Plastic	2.4	0.35
Titanium	105	0.31

**Solution stage**

**Figure 2.**
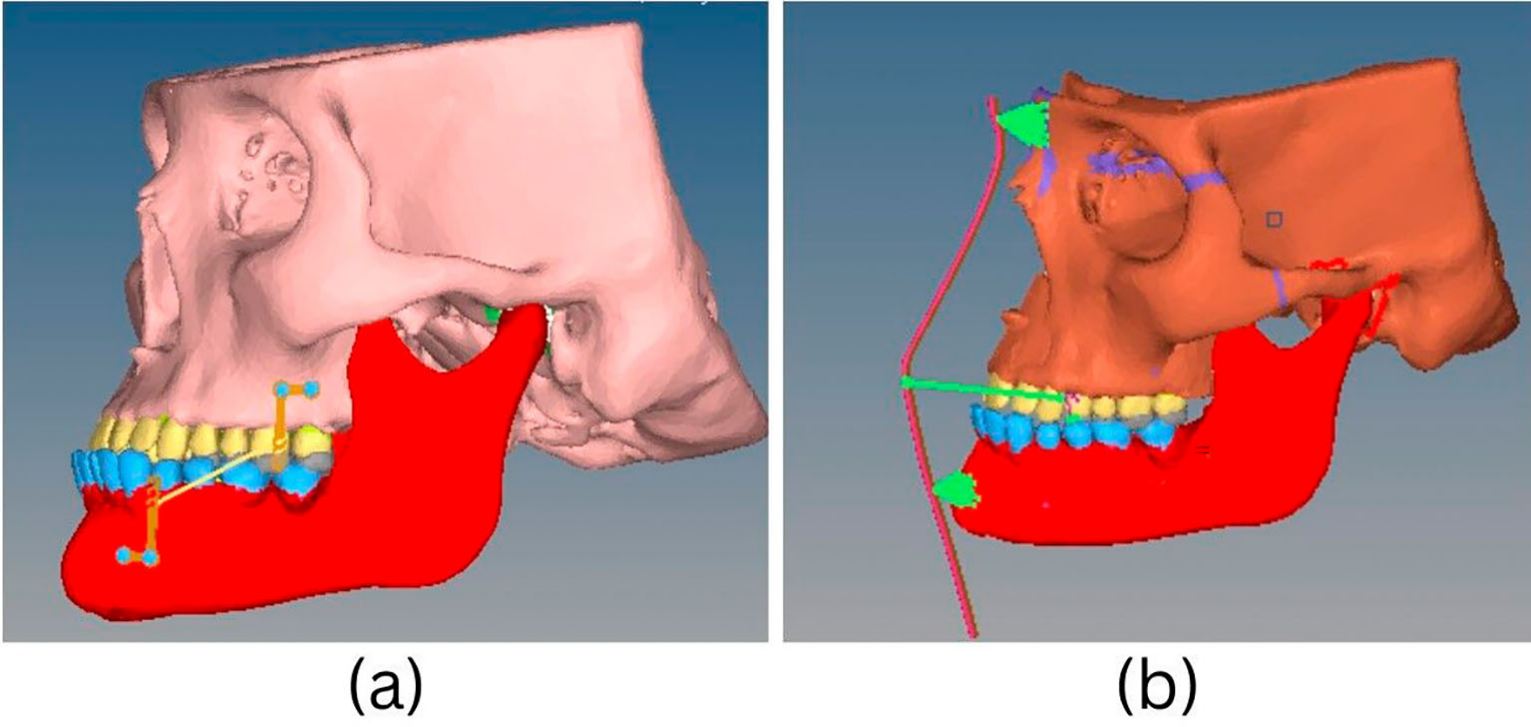
Lateral view of (a) BAMP and (b) Facemask protocol in ANSYS software for FEM analysis.

Determining boundary conditions and applying force are steps in the finite element analysis solution stage. The load and boundary conditions of the finite element model is described as follows:

Force loading - For BAMP and facemask (FM) therapy ( Figure 2), forces of 300 grams were applied to replicate different clinical protocols for maxillary protraction from the infrazygomatic buttress to the parasymphyseal area. The two procedures’ forces and their effects were examined.

Boundary conditions – Nodes in the cranial vault were restricted in all directions, with no rotation or displacement.

**Post-processing stage**

Areas of significant stress and displacement were depicted using contour plots using nodal and element solutions. The displacements along the X,Y, and Z planes have been presented. Also provided are Von Misses’ effective stresses.

## Results

### Displacement pattern

**Figure 3.**
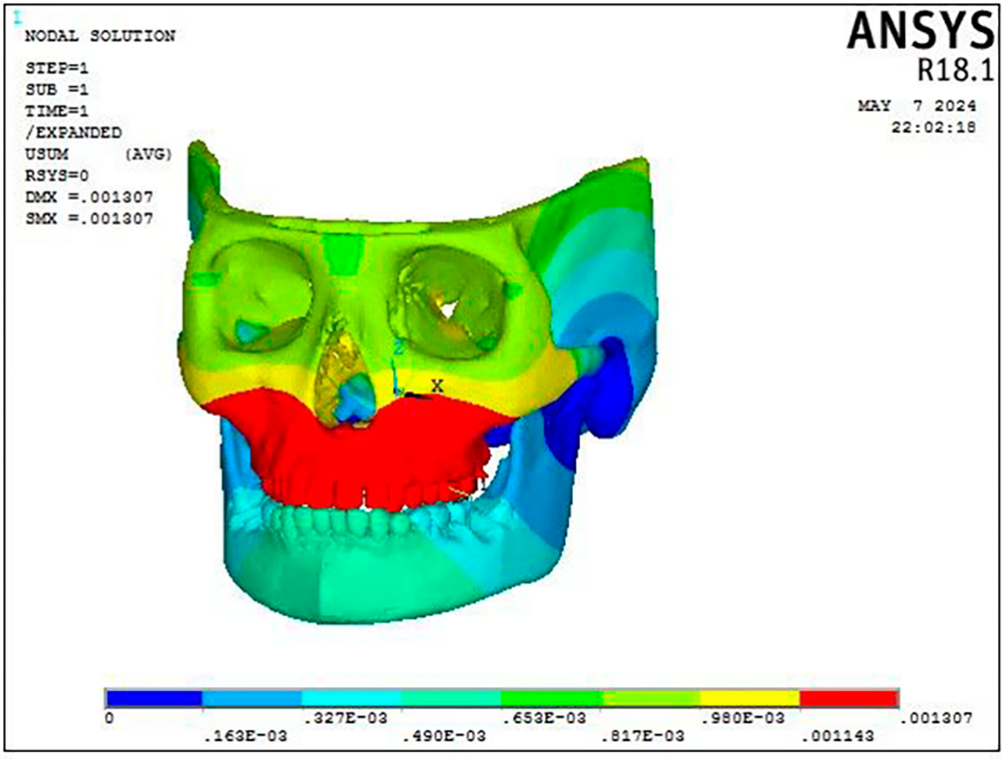
Displacement pattern of the craniofacial complex seen on application of 300 gm.

**Figure 4.**
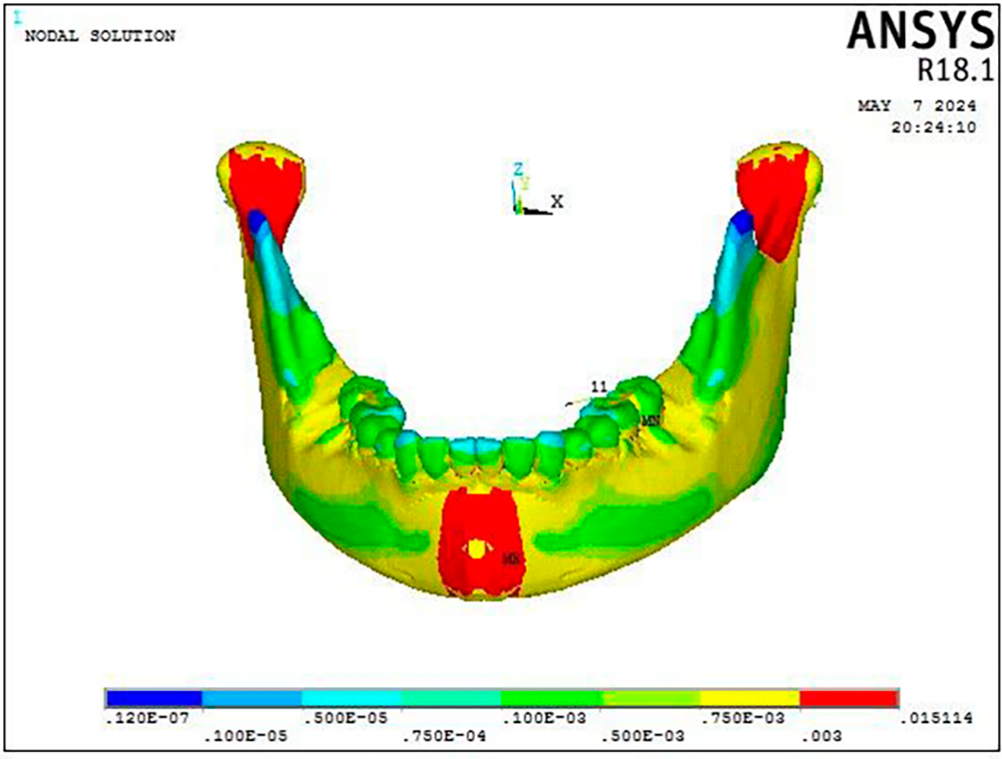
Displacement pattern of the mandible seen on the application of 300 gm force.

According to the results of the BAMP procedure, there is displacement across the craniofacial complex. As shown in Figure 3 and Table 2, the maxillary complex (0.001307 mm) and the dentoalveolar area (0.001307 mm) exhibit the greatest deformation in anterior direction. Mandibular displacement was noted in the condyle (0.000107 mm) and symphyseal area in a backward direction ( Figure 4).

**Table 2.** Displacement of craniofacial complex in BAMP and Facemask protocol simulation (mm).

	BAMP	FACEMASK
Overall deformation (mm)	0.001307	0.000105
Maxilla deformation (mm)	0.001307	0.000105
Mandible deformation (mm)	0.000107	0.0000283

**Figure 5.**
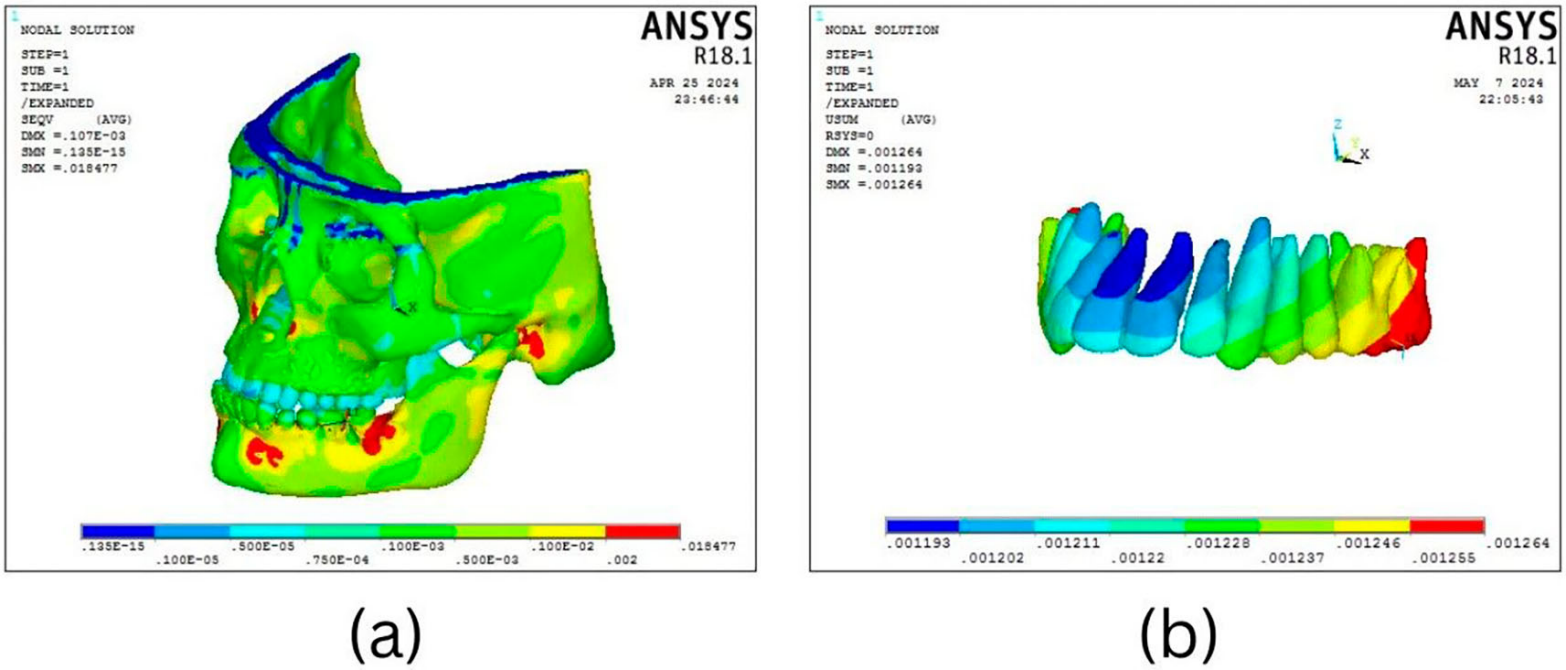
Overall stress distribution pattern of the (a) craniofacial complex and (b) maxillary dentition seen on the application of 300 gm force using BAMP protocol.

**Figure 6.**
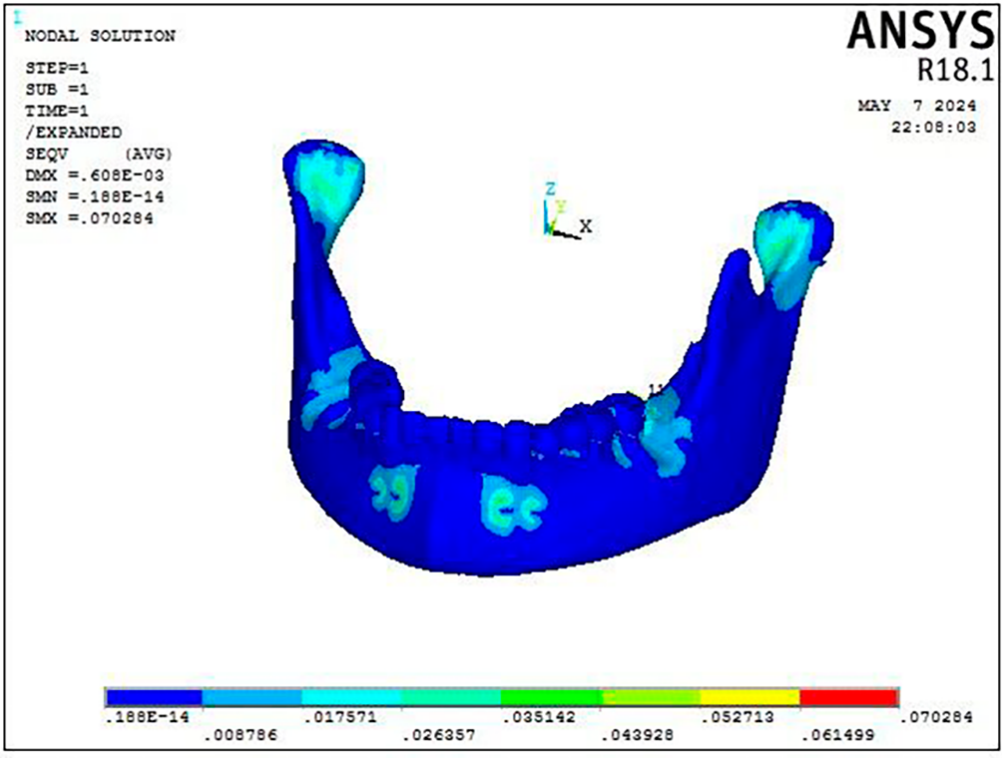
Stress distribution pattern of the mandible seen on the application of 300 gm force.

The Facemask protocol observations showed a discernible degree of deformation throughout the craniofacial complex, with the maxillary complex exhibiting the most significant deformation (0.000105 mm), particularly in the forward and downward direction as well as the dentoalveolar region ( Figure 5). As shown in Figure 6, the mandible showed the greatest displacement in a downward and backward direction (0.000283 mm) in the anterior dentition and symphysis area.

### Von misses stress distribution

**Figure 7.**
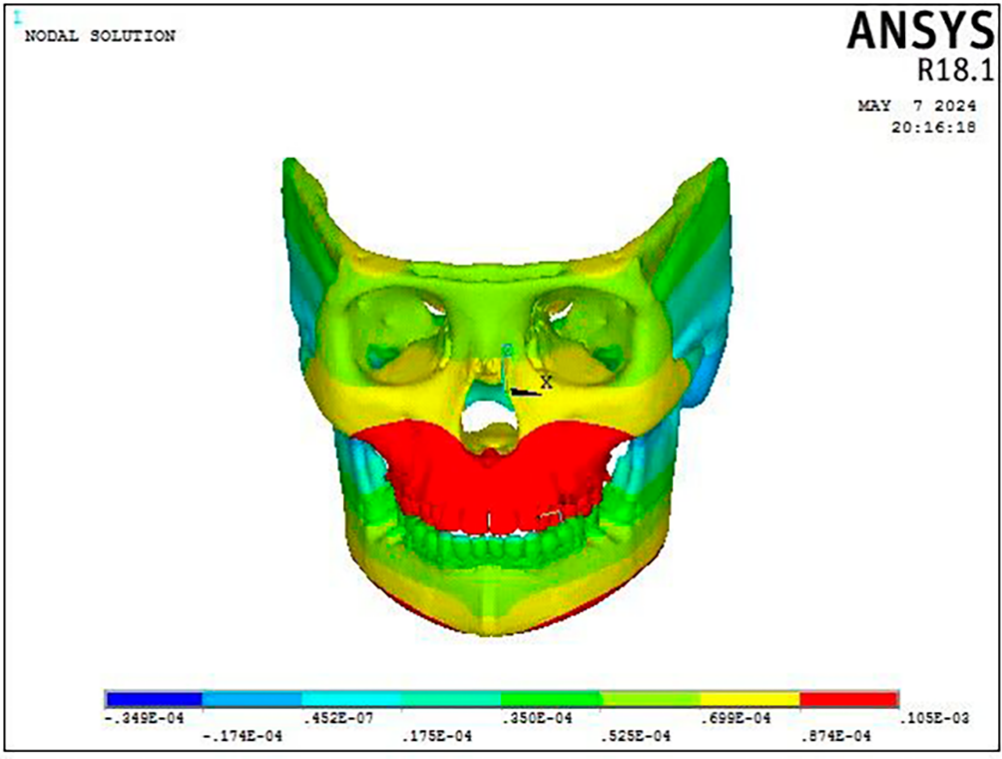
Displacement pattern of the craniofacial complex seen on application of 300 gm force.

**Figure 8.**
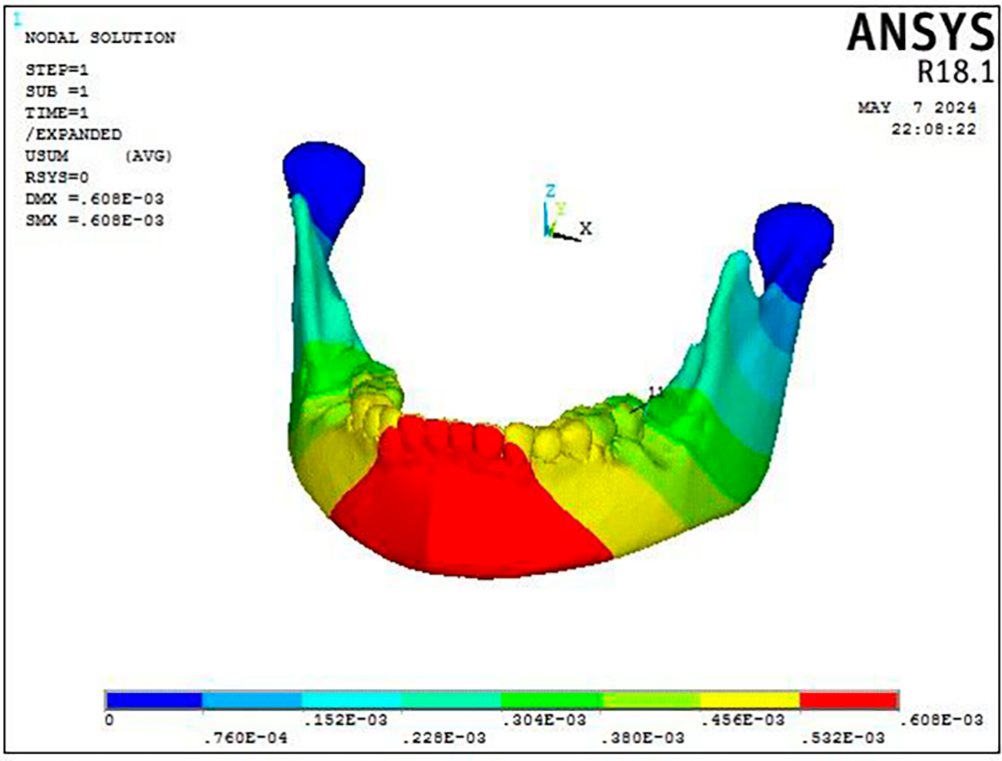
Displacement pattern of the mandible seen on the application of 300 gm force.

Stress was distributed throughout the entire craniofacial complex, with the miniplate attachment site showing the highest Von Misses stress at 0.95 MPa on mandible, as shown in Figure 4(a) and Table 3. The body of the mandible, zygomatic bone, maxillary dentition, and frontal bone showed the lowest stress, with values ranging from 0.013 MPa to 0.018 MPa. The parasymphyseal area and the condylar region exhibited the largest concentration of stress measuring 0.07 Mpa at the point of miniplate attachment ( Figure 7). The maxillary dentition had the highest Von Mises stress within the dentoalveolar complex, measuring 0.067 MPa at the miniplate attachment sites next to the first and second molars, in particular ( Figure 8).

**Table 3.** Von Misses stress distribution in BAMP and Facemask protocol simulation (MPa).

	BAMP	FACEMASK
Overall Stress (MPa)	0.950722	0.076198
Mandibular Stress (MPa)	0.070284	0.015114
Maxillary Stress (MPa)	0.950722	0.076198
Mandibular teeth Stress (MPa)	0.006985	0.004077
Maxillary teeth Stress (MPa)	0.001396	0.063745

**Figure 9.**
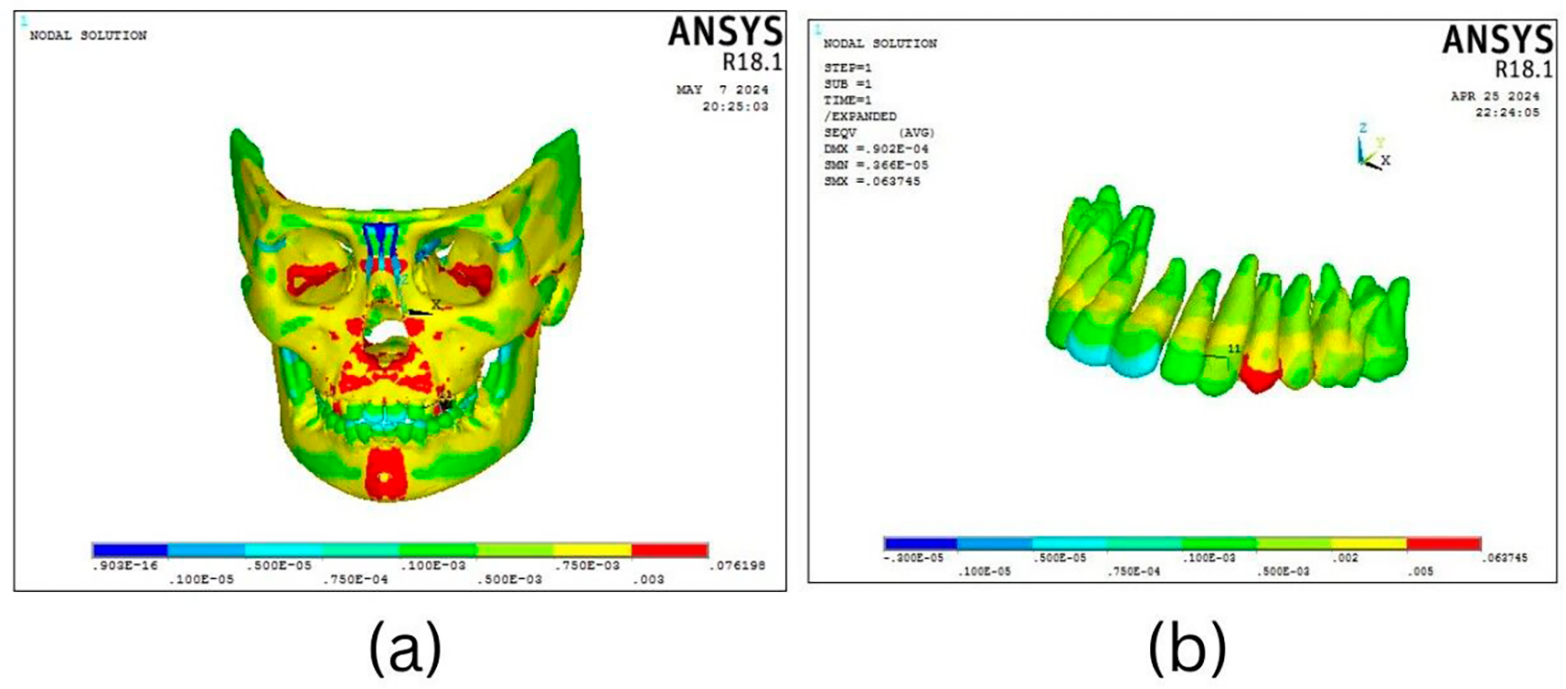
Stress distribution pattern of the (a) craniofacial complex and (b) maxillary dentition seen on the application of 300 gm force using Facemask protocol.

**Figure 10.**
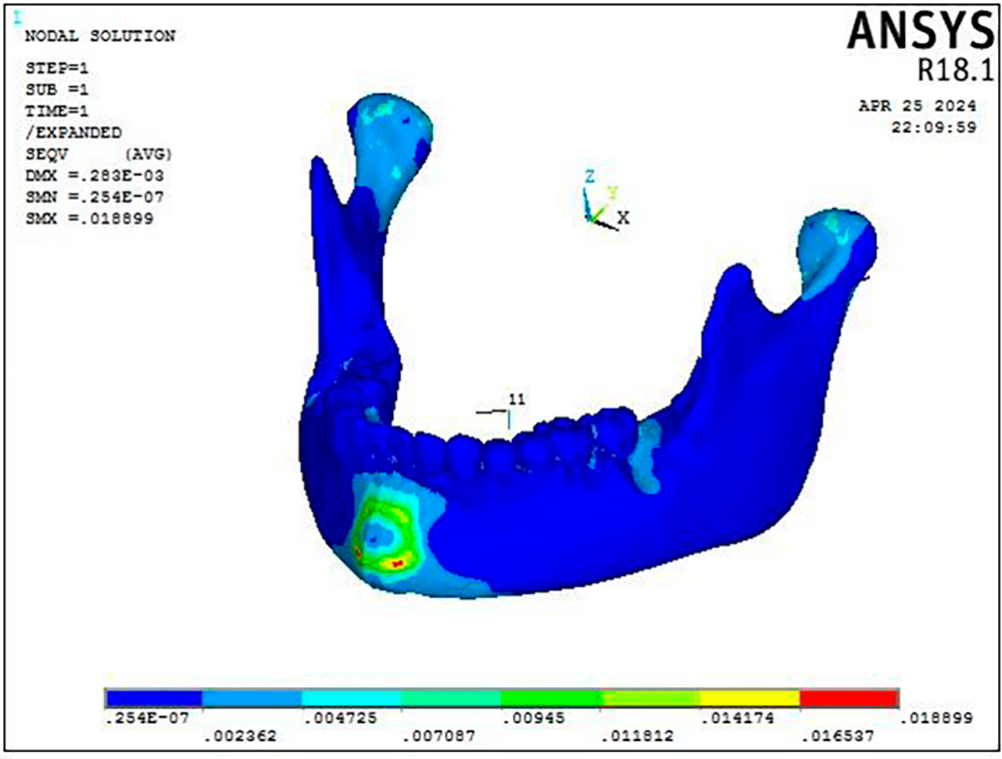
Stress distribution pattern of the mandible seen on the application of 300 gm force.

As illustrated in Figure 9(a), the FM protocol revealed stress distributions throughout the craniofacial complex, with the highest values at the frontonasal region, orbital area, mandibular symphysis, and maxillary region (0.07616 Mpa). The most significant stress within the mandible was concentrated at the site where the chin cup of the facemask was attached to the symphyseal area (0.015 MPa) with the rest of the structures exhibiting least stress ( Figure 10).

The maxillary dentition exhibited its highest Von Mises stress at the site of attachment for the facemask elastics, particularly in the premolar region measuring 0.0639 MPa, while the remaining teeth displayed relatively lower stress levels, especially notable in the crowns of the central incisors Figure 9(b).

## Discussion

This study aimed to evaluate and compare the displacement and stress distribution on various craniofacial structures using two orthodontic treatment protocols: Bone Anchored Maxillary Protraction (BAMP) and Facemask (FM), using the Finite Element Method (FEM). The BAMP protocol demonstrated significantly higher stress levels and displacement in the craniofacial structures compared to the Facemask protocol.

### Displacement effect on the maxilla

The maxillary complex exhibited greater deformation, particularly in the forward and downward directions, under both treatment protocols. The BAMP protocol demonstrated significantly superior maxillary advancement compared to the Facemask protocol. It effectively moved the zygoma, maxilla, and midface as a cohesive unit, highlighting its enhanced efficacy in promoting maxillary growth. ^8, 9^

The notable advancements achieved with the Bone Anchored Maxillary Protraction (BAMP) protocol can be attributed to its capacity to direct orthopedic forces specifically to the sutural sites of the maxilla through the use of skeletal anchorage. This direct application enhances the efficacy of maxillary advancement. In contrast, the Facemask (FM) treatment relies on tooth-borne anchorage, which results in incomplete transfer of orthopaedic forces to the sutures. Consequently, a significant portion of the applied force is dispersed to the periodontal ligament area, limiting the effectiveness of the treatment. ^10^

Another critical factor influencing the advancements seen with the Bone Anchored Maxillary Protraction (BAMP) protocol is patient compliance. BAMP demonstrates superior efficiency due to its reliance on minimal compliance requirements and the application of continuous forces. In contrast, the Facemask (FM) protocol depends on intermittent force application, which can be less effective if patient compliance is inconsistent. This difference in force application further underscores the advantages of BAMP in achieving consistent maxillary advancement. ^11^

### Displacement effect on the mandible

According to the present study, the Facemask (FM) protocol resulted in greater downward displacement of the mandible compared to the Bone Anchored Maxillary Protraction (BAMP) protocol. The existing literature presents conflicting conclusions regarding mandibular displacement. Some studies indicate that both the FM and BAMP protocols lead to clockwise rotation of the mandible. ^8, 12^ Other research suggests that only the FM protocol induces clockwise rotation, while the BAMP protocol is associated with counterclockwise rotation. ^9, 13^ These differing findings highlight the complexity of mandibular responses to treatment protocols.

In the Facemask (FM) protocol, the force exerted by the chin cup is thought to play a crucial role in redirecting mandibular growth downward and backward. This mechanical action not only influences mandibular positioning but also affects maxillary molar dynamics. Research indicates that the FM protocol is associated with the extrusion of the maxillary molars, which contributes to the observed clockwise rotation of the mandible during treatment. ^9, 14^

According to Bachetti et al. ^14^ the decrease in clockwise rotation of the mandible observed with the Bone Anchored Maxillary Protraction (BAMP) protocol is attributed to the horizontal backward movement of the mandible. This movement is accompanied by the relocation of the condyle within the glenoid fossa, which helps prevent mandibular backward rotation.

### Displacement effect on the dentoalveolar complex

The present study revealed that the Facemask (FM) protocol led to distinct changes in both the maxillary and mandibular dentitions. The maxillary dentition exhibited anterior displacement in both the molar and incisor regions and the mandibular dentition showed retroclination of the incisors. De Souza et al. ^15^ attributed the dentoalveolar side effects observed in the FM group to the reliance on a tooth-supported anchorage system. They proposed that proclination of upper incisors was a result of mesial dental movement. The uprighting of lower incisors has occurred due to pressure exerted by the chin cup and surrounding soft tissue.

The Bone Anchored Maxillary Protraction (BAMP) protocol resulted in noticeable displacement in the molar region of the maxilla. These findings are consistent with the study by Ngan et al. ^11^ which indicated that the maxillary molars moved forward despite the anchorage provided by the two mini-implants used in the BAMP protocol. In contrast, various studies ^9, 10, 16^ have reported that both the BAMP and Facemask protocols resulted in similar movements of the maxillary dentition, suggesting that while BAMP can achieve specific advancements, the overall effects may align with those observed with traditional Facemask therapy.

### Stress distribution on craniofacial complex

The overall stress distribution analysis of the craniofacial complex revealed that the Bone Anchored Maxillary Protraction (BAMP) protocol exhibited higher Von Mises stress levels compared to the Facemask (FM) protocol. High stress concentrations were observed in the parasymphyseal region, temporal bone and the lower border of the piriform aperture. The Von Mises stress distribution analysis for the Facemask (FM) protocol indicated that the highest stress concentrations were found in the symphyseal area of the mandible, maxilla and sphenoid bone.

The observed stress distribution pattern within the craniomaxillary complex correlates with the displacement patterns recorded in the study. This relationship underscores the biomechanical interactions during treatment. The study further suggests that sutures play a crucial role in craniofacial growth. It is proposed that exogenous forces applied to the maxilla are transmitted to distant structures within the craniofacial region through these sutures, influencing overall craniofacial development. ^16^

In the present study, the von Mises stress observed in all circummaxillary sutures was significantly higher in the Bone Anchored Maxillary Protraction (BAMP) protocol compared to the Facemask (FM) protocol. This increased stress suggests that greater bone remodeling occurs within the sutures during BAMP treatment. This enhanced remodeling elucidates the heightened displacement of the nasomaxillary complex achieved through protraction with skeletal anchorage. ^17– 19^

A major limitation of this study is that the results were derived from a three-dimensional finite element model. While this model demonstrates similar behaviour to actual biological structures, it does not fully replicate clinical scenarios. In a clinical environment, various factors can influence outcomes, such as variations in craniofacial anatomy among individuals, biological responses to treatment and patient compliance and other practical considerations. Given these factors, caution should be exercised when generalizing these findings to broader populations.

The study utilized a 300-gram force applied bilaterally, which may not accurately reflect the ideal force levels for either the Bone Anchored Maxillary Protraction (BAMP) or Facemask (FM) protocols. The present study acknowledges the need for further research to explore the effects of varying force levels on both protocols. Investigating a range of forces will help to more accurately measure displacement and stress distribution, ultimately leading to a better understanding of optimal treatment parameters.

## Conclusions

This study utilized Finite Element Method (FEM) to compare stress distribution and displacement between the Bone Anchored Maxillary Protraction (BAMP) and Facemask (FM) protocols for treating Class III malocclusion. The following conclusions can be drawn:

• The BAMP protocol exhibited greater stress and displacement in the craniofacial region compared to the Facemask protocol.
• Both protocols resulted in downward and backward rotation of the mandible, with the Facemask group displaying a higher degree of displacement.
• In the Facemask protocol, the anterior teeth demonstrated increased proclination, while the mandibular anterior teeth showed retroclination. In contrast, the maxillary dentoalveolar complex in the BAMP protocol exhibited significant anterior displacement in the first and second molar regions, indicating mesialization of the molars.
• Stress distribution throughout the craniofacial complex was observed in both protocols, with BAMP showing higher stress levels. This suggests effective transfer of stresses throughout the complex, facilitating sutural disarticulation.

## Ethics and consent

The protocol was approved by the Institutional Review Board of College of Dental Sciences, India (Approval number: CODS/IEC/32/2021-22). The Committee on Research Ethics followed internationally recognized guidelines for the protection of human subjects, including the Declaration of Helsinki, the Belmont Report, and CIOMS principles. Prior to participation, all individuals received a clear explanation of the study objectives, procedures, and intended use of their craniofacial images for finite element modeling. Written informed consent was obtained from the subject involved in this study before the commencement of the study.

## Data Availability

Figshare – Assessment of Stress Distribution and Displacement in the Craniofacial Complex between Facemask Therapy and Bone Anchored Maxillary Protraction (BAMP): A 3D Finite Element Study. https://doi.org/10.6084/m9.figshare.30631322.v1. ^20^ This project contains following underlying data:
• study-data.xlsx Data are available under the terms of the Creative Commons Zero “No rights reserved” data waiver (CC BY 4.0 Public domain dedication).
