# Supplementary material for: Assessment of Stress Distribution and Displacement in the Craniofacial Complex between Facemask Therapy and Bone Anchored Maxillary Protraction (BAMP): A 3D Finite Element Study
Source: F1000Res. 2025 Dec 22;14:1429. [Version 1] doi: 10.12688/f1000research.173765.1 (PMC12895103; doi:10.12688/f1000research.173765.1)
Supplement: Peer review history

# Reviewer report: Reviewer response for version 1

**DOI:** 10.5256/f1000research.191608.r453085

**Published:** 2026-02-11

**Authors:** Luz Andrea Velandia Palacio

**Affiliations:** 1 CICO, Institución Universitaria Colegios de Colombia UNICOC, Bogotá, Colombia

Dr. Saji and colleagues addressed an important topic by comparing facemask therapy (FM) and bone-anchored maxillary protraction (BAMP) using three-dimensional finite element analysis. Their study focuses on stress distribution and displacement within the craniofacial complex, providing important insight into the biomechanical responses associated with the two well established therapies for Class III correction.

 The results of the model indicated that maxillary protraction was achieved with both methods. However, greater dental effects were observed with FM, along with more pronounced mandibular downward displacement compared to BAMP, suggesting that BAMP delivers a more directly skeletal force to the maxillary complex with reduced dentoalveolar effects and less posterior displacement of the mandible, thereby limiting an increase in vertical dimension. Consequently, the findings indicate that BAMP may be superior for the treatment of Class III malocclusion.  The article is well written and presents clear data illustrating the distinct patterns of stress distribution and displacement associated with each method. Nevertheless, several inconsistencies hinder the accurate interpretation of the results.

 First, there appears to be a mismatch between the text and the figures. Several figures described in the manuscript as depicting von Mises stress instead appear to present nodal displacement (deformation). For example, Figure 8 is referenced as showing von Mises stress in the maxillary dentition, but the corresponding image displays a displacement pattern of the mandible. Figure 7 also appears to be missing. The authors should verify all figure citations and ensure that each figure presents the correct result and is properly referenced.

 Second, the manuscript reports directional displacements (forward, downward, backward), but the provided illustrations show only total deformation. As directional movement cannot be inferred from total displacement maps, it is recommended that the authors present directional displacement components (such as Ux and Uy) or vector plots to clarify the coordinate system orientation.

 A third minor issue is a typographical error in the Discussion section, where the name “Baccetti” is misspelled. This should be corrected throughout the article to ensure accurate referencing.

 Overall, this article contributes with important information that can guide clinical decision making for Class III treatment. However, clarification of figure labeling, result types, and directional analysis is necessary to improve clarity and to fully support the significant findings presented.

Is the work clearly and accurately presented and does it cite the current literature?

Yes

If applicable, is the statistical analysis and its interpretation appropriate?

Not applicable

Are all the source data underlying the results available to ensure full reproducibility?

Yes

Is the study design appropriate and is the work technically sound?

Yes

Are the conclusions drawn adequately supported by the results?

Partly

Are sufficient details of methods and analysis provided to allow replication by others?

Yes

Reviewer Expertise:

Orthodontics and Dentofacial OrthopedicsDigital Orthodontics and 3D WorkflowsClear Aligner Therapy and BiomechanicsCraniofacial Growth and MaturationForensic and Dental Age Estimation

I confirm that I have read this submission and believe that I have an appropriate level of expertise to confirm that it is of an acceptable scientific standard, however I have significant reservations, as outlined above.

# Reviewer report: Reviewer response for version 1

**DOI:** 10.5256/f1000research.191608.r453089

**Published:** 2026-02-09

**Authors:** Fuad Lutf Almotareb

**Affiliations:** 1 Sana'a University, Sana'a, Capital Municipality, Yemen

**Title:** “Assessment of Stress Distribution and Displacement in the Craniofacial Complex between Facemask Therapy and Bone Anchored Maxillary Protraction (BAMP): A 3D Finite Element Study”

 This manuscript addresses a clinically relevant orthodontic topic; however, important methodological limitations restrict biological interpretation of the findings.

**Major Concerns**

**1. Biological Validity of the Finite Element Model**

 “The manuscript states that FEA was used to evaluate biomechanical effects in the craniofacial complex. “

**Comment:** The methodology employs a dry skull model lacking sutures and a periodontal ligament (PDL). Such a model cannot reliably represent biologically relevant orthopedic response. Therefore, the findings should be interpreted strictly as simplified mechanical stress/displacement patterns rather than true craniofacial biomechanics.

**2. Description of the Sample**

 “The manuscript describes the model as a “young human skull.”

**Comment:** The use of the term "young human skull"  is potentially misleading.

 The description should be revised to accurately reflect that the model represents a geometric skull structure rather than an age-specific biological specimen.

**3. Interpretation of Skeletal vs Dental Effects**

 "Greater skeletal displacement" and "Fewer dental effects"

**Comment:** The conclusion that BAMP produces greater skeletal displacement with fewer dental effects is not sufficiently supported by the modeling conditions. The conclusions should therefore be substantially moderated and limited to comparative mechanical behavior within the constraints of the model.

**4. Force Application Protocol**

 “An identical force magnitude (300 g) and direction were applied for both facemask and BAMP protocols.”

**Comment:** This does not reflect clinical biomechanics, where force magnitude, point of application, and vector differ significantly between the two modalities. The force protocol must be justified separately for each technique.

**Minor Concerns**

**Terminology:** Replace “Von misses” with Von Mises stress

**Statistical Reporting**: The manuscript refers to “statistical significance” despite the absence of statistical testing.

 This term should be removed or replaced with descriptive comparison.

**Organization of Results**: The Results section would benefit from clearer structure:

 First by treatment modality (Facemask vs BAMP)

 Then by outcome variable (displacement vs stress distribution)

**Language and Formatting**: Use past tense consistently in Methods and Results, Standardize units to MPa

 Verify all figure and table citations for accuracy

**Recommendation:   **Requires major revisions

 Thanking you

 Dr. Fuad Almotareb

Is the work clearly and accurately presented and does it cite the current literature?

Partly

If applicable, is the statistical analysis and its interpretation appropriate?

I cannot comment. A qualified statistician is required.

Are all the source data underlying the results available to ensure full reproducibility?

Partly

Is the study design appropriate and is the work technically sound?

Yes

Are the conclusions drawn adequately supported by the results?

Yes

Are sufficient details of methods and analysis provided to allow replication by others?

Partly

Reviewer Expertise:

orthodontic

I confirm that I have read this submission and believe that I have an appropriate level of expertise to confirm that it is of an acceptable scientific standard, however I have significant reservations, as outlined above.

# Reviewer report: Reviewer response for version 1

**DOI:** 10.5256/f1000research.191608.r445578

**Published:** 2026-01-07

**Authors:** Goutham B

**Affiliations:** 1 Department of Orthodontics and Dentofacial Orthopaedics, Coorg Institute of Dental Sciences, Virajpet, Karnataka, India

The article is well written. Appropriate methodology has been followed. The article effectively assesses changes in the craniofacial complex between two most commonly used methods for maxillary protraction and ensures that readers gain an understanding of effect of these techniques including stress distribution. The authors not only summarize existing studies but also highlight limitations which adds depth and balance to the discussion section. The discussion provides valuable guidance for future research.

Is the work clearly and accurately presented and does it cite the current literature?

Yes

If applicable, is the statistical analysis and its interpretation appropriate?

I cannot comment. A qualified statistician is required.

Are all the source data underlying the results available to ensure full reproducibility?

Yes

Is the study design appropriate and is the work technically sound?

Yes

Are the conclusions drawn adequately supported by the results?

Yes

Are sufficient details of methods and analysis provided to allow replication by others?

Yes

Reviewer Expertise:

orthodontics

I confirm that I have read this submission and believe that I have an appropriate level of expertise to confirm that it is of an acceptable scientific standard.
